# Short Physical Performance Battery and all-cause mortality: systematic review and meta-analysis

**DOI:** 10.1186/s12916-016-0763-7

**Published:** 2016-12-22

**Authors:** Rita Pavasini, Jack Guralnik, Justin C. Brown, Mauro di Bari, Matteo Cesari, Francesco Landi, Bert Vaes, Delphine Legrand, Joe Verghese, Cuiling Wang, Sari Stenholm, Luigi Ferrucci, Jennifer C. Lai, Anna Arnau Bartes, Joan Espaulella, Montserrat Ferrer, Jae-Young Lim, Kristine E. Ensrud, Peggy Cawthon, Anna Turusheva, Elena Frolova, Yves Rolland, Valerie Lauwers, Andrea Corsonello, Gregory D. Kirk, Roberto Ferrari, Stefano Volpato, Gianluca Campo

**Affiliations:** 1 Cardiology Unit, Azienda Ospedaliero-Universitaria S.Anna, Via Aldo Moro, 8, 44124 Ferrara, Italy; 2 University of Maryland School of Medicine, Baltimore, MD USA; 3 Division of Population Sciences, Department of Medical Oncology, Dana-Farber Cancer Institute, Boston, MA USA; 4 Department of Critical Care Medicine and Surgery, Unit of Gerontology and Geriatric Medicine, University of Florence and Azienda Ospedaliero-Universitaria Careggi, Florence, Italy; 5 Gérontopôle, Centre Hospitalier Universitaire de Toulouse, Toulouse, France; 6 INSERM UMR1027, Université de Toulouse III Paul Sabatier, Toulouse, France; 7 Department of Geriatrics, Neurosciences and Orthopaedics, Catholic University of the Sacred Heart, Rome, Italy; 8 Institute of Health and Society, Université catholique de Louvain (UCL), Brussels, Belgium; 9 Department of Public Health and Primary Care, KU Leuven (KUL), Leuven, Belgium; 10 Institut de Recherche Sante et Societe, Universite Catholique de Louvain, Brussels, Belgium; 11 Department of Neurology & Medicine, Albert Einstein College of Medicine, Bronx, New York USA; 12 Department of Epidemiology, Albert Einstein College of Medicine, Bronx, New York USA; 13 Department of Public Health, University of Turku, Turku, Finland; 14 National Institute on Aging, Longitudinal Studies Section, Clinical Research Branch, NIA-ASTRA Unit, Harbor Hospital, Baltimore, MD USA; 15 Department of Medicine, Division of Gastroenterology and Hepatology, University of California San Francisco, San Francisco, CA USA; 16 Clinical Research Unit, Althaia Xarxa Assistencial Universitària de Manresa, Manresa, Barcelona Spain; 17 Servei de Geriatria i Cures Palliatives, Hospital Universitari de la Santa Creu, Vic, Barcelona Spain; 18 Health Services Research Group, IMIM, Hospital del Mar Medical Research Institute, Barcelona, Spain; 19 CIBER Epidemiología y Salud Pública (CIBERESP), Barcelona, Spain; 20 Department of Rehabilitation Medicine, Seoul National University Bundang Hospital, Seoul National University College of Medicine, Seongnam, Gyeonggi Republic of Korea; 21 Medicine and Epidemiology & Community Health, University of Minnesota, Minneapolis, Minnesota USA; 22 General Internal Medicine, Minneapolis VA Health Care System, Minneapolis, Minnesota USA; 23 California Pacific Medical Center Research Institute, San Francisco, CA USA; 24 Department of Family Medicine, The North-Western State Medical University named after I.I. Mechnikov, St. Petersburg, Russia; 25 Faculte’ de Me’ decine, Laboratoire d’Epidemiologie et Sante’ Communautaire (Unite’ Inserm 558), Alle’es Jules Guesde, Toulouse, France; 26 Unit of Geriatric Pharmacoepidemiology, Research Hospital of Cosenza, Italian National Research Centre on Aging (INRCA), Cosenza, Italy; 27 Departments of Epidemiology and Medicine, Johns Hopkins University, Baltimore, MD USA; 28 Maria Cecilia Hospital, GVM Care & Research, E.S.: Health Science Foundation, Cotignola, Italy; 29 Department of Medical Science, Section of Internal and Cardiorespiratory Medicine, University of Ferrara, Ferrara, Italy

**Keywords:** Short Physical Performance Battery, All-cause mortality, Physical function, Meta-analysis

## Abstract

**Background:**

The Short Physical Performance Battery (SPPB) is a well-established tool to assess lower extremity physical performance status. Its predictive ability for all-cause mortality has been sparsely reported, but with conflicting results in different subsets of participants. The aim of this study was to perform a meta-analysis investigating the relationship between SPPB score and all-cause mortality.

**Methods:**

Articles were searched in MEDLINE, the Cochrane Library, Google Scholar, and BioMed Central between July and September 2015 and updated in January 2016. Inclusion criteria were observational studies; >50 participants; stratification of population according to SPPB value; data on all-cause mortality; English language publications. Twenty-four articles were selected from available evidence. Data of interest (i.e., clinical characteristics, information after stratification of the sample into four SPPB groups [0–3, 4–6, 7–9, 10–12]) were retrieved from the articles and/or obtained by the study authors. The odds ratio (OR) and/or hazard ratio (HR) was obtained for all-cause mortality according to SPPB category (with SPPB scores 10–12 considered as reference) with adjustment for age, sex, and body mass index.

**Results:**

Standardized data were obtained for 17 studies (*n* = 16,534, mean age 76 ± 3 years). As compared to SPPB scores 10–12, values of 0–3 (OR 3.25, 95%CI 2.86–3.79), 4–6 (OR 2.14, 95%CI 1.92–2.39), and 7–9 (OR 1.50, 95%CI 1.32–1.71) were each associated with an increased risk of all-cause mortality. The association between poor performance on SPPB and all-cause mortality remained highly consistent independent of follow-up length, subsets of participants, geographic area, and age of the population. Random effects meta-regression showed that OR for all-cause mortality with SPPB values 7–9 was higher in the younger population, diabetics, and men.

**Conclusions:**

An SPPB score lower than 10 is predictive of all-cause mortality. The systematic implementation of the SPPB in clinical practice settings may provide useful prognostic information about the risk of all-cause mortality. Moreover, the SPPB could be used as a surrogate endpoint of all-cause mortality in trials needing to quantify benefit and health improvements of specific treatments or rehabilitation programs.

The study protocol was published on PROSPERO (CRD42015024916).

**Electronic supplementary material:**

The online version of this article (doi:10.1186/s12916-016-0763-7) contains supplementary material, which is available to authorized users.

## Background

Life expectancies at birth have risen globally, with the longest life expectancies (80–87 years) in Europe and North America [1]. With this has come the challenge of providing medical care to increasingly older adults. It is well established that the elderly are at increased risk of frailty, functional decline, and other adverse health outcomes, as well as death [1, 2]. This finding has important clinical implications, because impaired functional status significantly influences prognosis and benefit from pharmacological and interventional therapies. As such, several authors and experts have suggested that the assessment of physical performance and functional status should be included in the initial clinical evaluation of older patients [3], with the aim of guiding clinicians in the decision-making process. The Short Physical Performance Battery (SPPB) has emerged as one of the most promising tools to evaluate functional capability and provide a measure of the biological age of an older individual [4]. It is an objective tool for measuring the lower extremity physical performance status [4]. The SPPB is based on three timed tasks: standing balance, walking speed, and chair stand tests. The timed results of each subtest are rescaled according to predefined cut-points for obtaining a score ranging from 0 (worst performance) to 12 (best performance) [4]. The SPPB has been adopted in multiple observational studies that have consistently found an association with incident disability and hospital admission [3, 28]. Some studies suggest SPPB also has the capacity to predict all-cause mortality [6–29]. However, results were inconclusive, perhaps due to (1) limited sample size, (2) heterogeneous cut-points for categorizing the timed results, and (3) variability in the clinical settings of applications [6–29].

Therefore, the aim of this study was to assess the relationship between SPPB and all-cause mortality by performing a thorough systematic review and meta-analysis.

## Methods

We developed a systematic review and meta-analysis following the Preferred Reporting Items for Systematic Review and Meta-Analyses (PRISMA) amendment to the Quality of Reporting of Meta-analyses (QUOROM) statement and recommendations from the Cochrane Collaboration and from the Meta-analysis of Observational Studies in Epidemiology (MOOSE) [30–33]. The protocol was previously published in an international prospective register of systematic reviews (PROSPERO) under number CRD42015024916.

### Search strategy

Appropriate articles were found using the Medical Subject Headings (MeSH) strategy and searching in MEDLINE, the Cochrane Library, Google Scholar, and BioMed Central. The search strategy was created by RP. The terms searched were: ((short physical performance battery) OR (SPPB) OR (lower limb strength) OR (standing balance) OR (walking speed) OR (chair stand)) AND ((mortality) OR (death)).

Only articles published in the English language and in peer-reviewed journals were selected. The research was carried out between July 2015 and January 2016. Independent reviewers (RP, GC) analyzed the titles and abstracts of the articles and determined which of them warranted the examination of the full text. Studies included in the analysis had to have the following characteristics: (1) observational (non-randomized) study; (2) inclusion of more than 50 subjects; (3) reporting the stratification of patients/population according to SPPB cut-points; (4) presenting data on all-cause mortality in relation to the value of SPPB expressed as hazard ratio (HR) or odds ratio (OR). Duplicate, interventional, or animal studies were excluded. Both reviewers agreed to the final number of studies included in the present analysis.

### Data extraction, definition, endpoint, and contact with authors

**Fig. 1:**
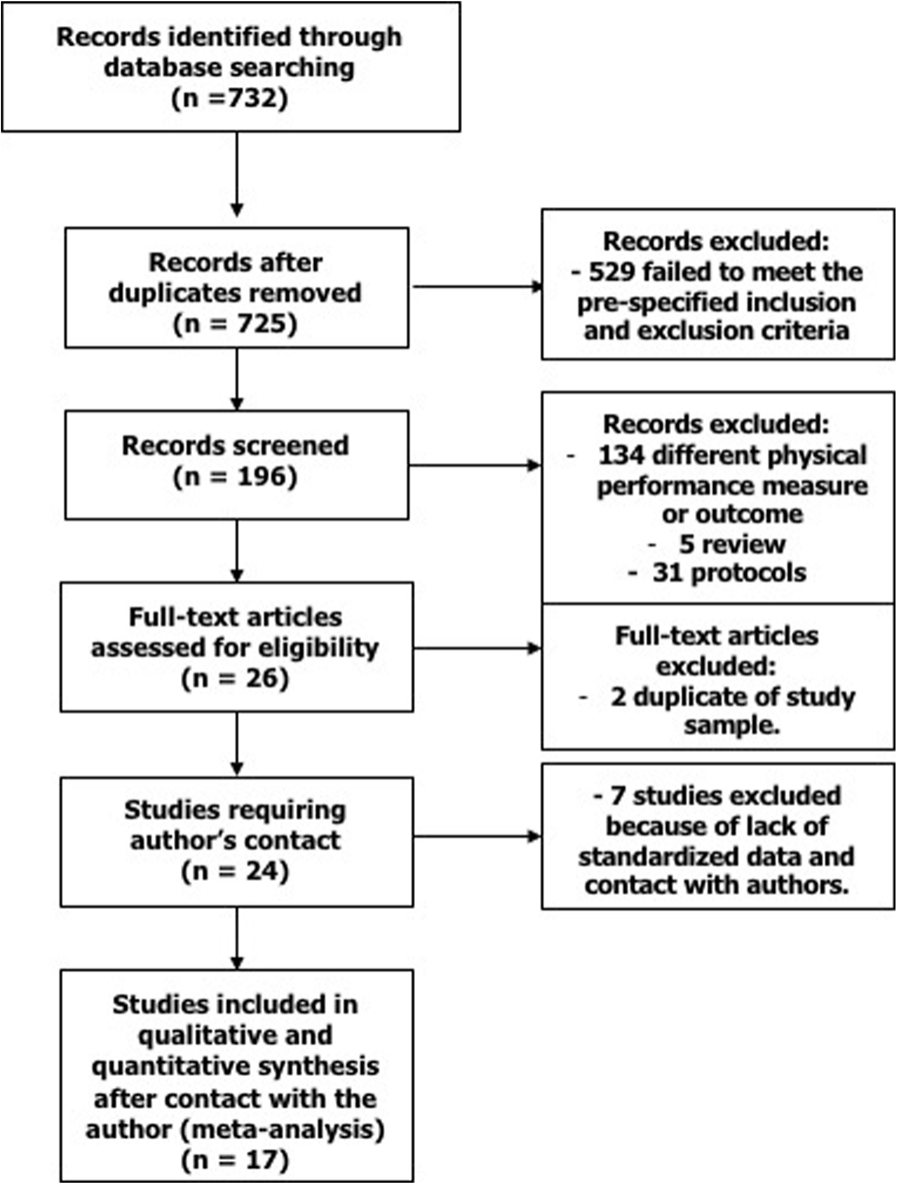
Search strategy. *n* number

Independent reviewers (GC, RP, and SV) completed the database, which contained information about the journal, year of publication, authors, baseline characteristics of study population, follow-up length, SPPB cut-points, and source of mortality data. The primary endpoint was all-cause mortality. Additional analyses were performed after stratification of studies according to the following criteria: (1) mean age of the study population (≤75 years versus >75 years); (2) setting (general population versus outpatients versus hospitalized patients); (3) geographical region (North America versus Europe versus Asia); (4) follow-up length (≤1 year versus >1 year and ≤5 years versus >5 years). To obtain standardized data, the authors of all the selected papers (*n* = 24) were contacted. Of the 22 authors contacted (two were corresponding authors for two studies), one was not able to provide the requested data, one refused to participate, and five never replied to the inquiry. A total of 15 authors (68%) gave complete available data for 17 of the studies originally selected (71%) (see Fig. 1). Authors were asked to complete a table summarizing baseline characteristics of their studies (mean age, sex, hypertension, cardiovascular disease, cerebrovascular disease, diabetes) and to stratify the population into four SPPB score categories (0–3, 4–6, 7–9, 10–12) according to the cut-points provided by Guralnik and colleagues in their original work [4]. The reference group for the analyses comprised participants ranging between 10 and 12 on the SPPB score. In addition, authors were asked to calculate the odds ratio (OR)/hazard ratio (HR) for all-cause mortality in SPPB groups with values 0–3, 4–6, and 7–9 compared to the group 10–12 as reference, and to perform multivariate analyses adjusted for age, sex, and body mass index (weight/height^2^).

### Internal validity and quality appraisal

Two unblinded reviewers (RP and SV) independently evaluated the quality of the included studies using prespecified electronic forms (piloted over the first three cases) and a modified version of the Newcastle-Ottawa Scale (NOS) for cohort studies [34] (Additional file 1: eTable 1). Because of the design of the studies considered, we did not consider the section for “Comparability” and question 2 in the section “Selection” (“selection of the non exposed cohort”). Discrepancies between reviewers were solved by consensus. No study was excluded on the basis of this analysis. The same reviewers independently analyzed references of all the evaluated articles to avoid the eventual exclusion of additional studies.

### Data analysis and synthesis

Continuous variables were reported as mean (± standard deviation) or median (interquartile range). Categorical variables were expressed as number and percentage (%). Point estimates and standard errors were extracted from individual studies and combined by the generic inverse variance method [35], computing risk estimates with 95% confidence intervals according to logarithmic transformation of the hazard measures. Considering the high likelihood of between-study variance, a random effect model was used. Statistical heterogeneity was assessed using Cochran’s Q test. This statistic was complemented with the *I* ^2^ statistic, which quantifies the proportion of total variation across studies that is due to heterogeneity rather than chance. A value for *I* ^2^ of 0–25% represents insignificant heterogeneity, 26–50% low heterogeneity, 51–75% moderate heterogeneity, and >75% high heterogeneity [36]. The chi-square test was used to test differences between subgroups. To estimate the percentage of deaths that could be attributed to poor physical function, the percentage attributable risk (%AR) was calculated [37]. Finally, a random effect meta-regression analysis was performed to assess the effect of some potential confounding factors (age, sex, previous history of cardiovascular disease, previous history of cerebrovascular disease, diabetes, hypertension) on the results. Publication bias was appraised by graphical evaluation of funnel plots and through Begg and Mazumdar rank correlation, Egger’s regression intercept, and Duval and Tweedie trim and fill [36]. Statistical analyses were conducted using ProMeta software (Internovi, Cesena, Italy) and RevMan 5 (the Cochrane Collaboration, the Nordic Cochrane Centre, Copenhagen, Denmark).

## Results

### Search results and study selection

After removal of duplicates, 725 titles were identified by the databases search (Fig. 1). Overall, 529 items were excluded after the first evaluation of the title and abstract, as they failed to meet the prespecified inclusion and exclusion criteria. Of the remaining 196 records examined, 134 were excluded because they focused on other outcomes or on other physical performance measures. An additional 5 were not retained because they were not original papers but reviews, and 31 because they were study protocols. Twenty-six studies were examined as full papers. Two of these were excluded because they were based on the same study sample used in Lai et al. [15]. The corresponding authors of the retained 24 records were contacted [6–29]. As previously explained, standardized information was obtained for 17 of them [5–21], and these studies were included in the final qualitative and quantitative analysis (Fig. 1).

### Population characteristics

A total of 16,534 participants (mean age: 76 ± 3 years, women: 78%) were included in the meta-analysis. Overall, 47% of participants had hypertension, 9% diabetes, and 31% prior diagnosis of cardiovascular disease, while only 5% had a previous diagnosis of cerebrovascular disease (Table 1). Eleven studies were focused on the general population [7, 8, 12–14, 16–21]. Of note, the study of Greene et al. focused on current and former injection drug users [13]. Three studies enrolled hospital inpatients [10, 11, 22]. In the study by Volpato et al., the causes of hospitalization were congestive heart failure (HF), chronic obstructive pulmonary disease, pneumonia, or minor stroke [22]. In the study by Chiarantini et al., the cause of hospitalization was decompensated HF [10], whereas in the study by Corsonello et al., patients were admitted to an acute care medical ward or a long-term care/rehabilitation unit [11]. The remaining three studies were based on outpatients presenting heterogeneous clinical conditions (primary care center: cancer, cirrhosis, on waiting list for liver transplant) (Table 1) [6, 9, 15]. Eleven studies were performed in Europe [6, 8–11, 16–20, 22], five in America [7, 12, 13, 15, 21], and one in Asia [14] (Table 1). The SPPB score was 0–3 in 9% of the study population, 4–6 in 16%, 7–9 in 33%, and 10–12 in 42%, respectively. Quality assessment of the 17 studies according to the NOS demonstrated an average high quality level with all studies having a score ranging between 5 and 6 (Additional file 1: eTable 1).

**Table 1:** Sample characteristics of the studies included in the meta-analysis

References	*N*	Participants	Nation	Mean age ±SD (y)	Female (%)	Hypertension (%)	DM (%)	CVA (%)	CVD (%)
Arnau et al. 2016 [6]	315	Outpatients	Spain-Eu	82 ± 5	191 (61)	–	62 (20)	–	–
Brown et al. 2015 [7]	413	General population	USA-Am	73 ± 1	248 (60)	189 (46)	43 (10)	28 (7)	63 (15)
Cesari et al. 2008 [8]	200	General population	IT-Eu	86 ± 1	223 (67)	246 (73)	92 (27)	15 (4)	40 (12)
Cesari et al. 2013 [9]	335	Outpatients	IT-Eu	74 ± 6	200 (100)	166 (83)	33 (17)	2 (1)	40 (20)
Chiarantini et al. 2010 [10]	157	Hospitalized	IT-Eu	80 ± 1	79 (50)	52 (33)	–	–	77 (49)
Corsonello et al. 2012 [11]	506	Hospitalized	IT-Eu	80 ± 6	231 (46)	357 (71)	130 (26)	66 (13)	149 (29)
Ensrud et al. 2016 [12]	1495	General population	USA-Am	88 ± 3	1495 (100)	985 (66)	219 (15)	194 (13)	373 (25)
Greene et al. 2014 [13]	1627	General population^a^	USA-Am	49 ± 8	561 (34)	–	–	–	–
Kim et al. 2016 [14]	560	General population	Korea-As	74 ± 7	274 (49)	256 (46)	49 (9)	87 (16)	87 (16)
Lai et al. 2014 [15]	294	Outpatients	USA-Am	57 ± 10	100 (34)	123 (42)	85 (29)	4 (1)	17 (6)
Legrand et al. 2014 [16]	560	General population	BE-Eu	85 ± 4	351 (63)	393 (70)	104 (19)	46 (8)	140 (25)
Minneci et al. 2015 [17]	561	General population	IT-Eu	73 ± 1	323 (58)	–	–	–	–
Rolland et al. 2006 [18]	7250	General population	FR-Eu	81 ± 4	7250 (100)	3429 (47)	420 (6)	210 (3)	3495 (48)
Stenholm et al. 2016 [19]	996	General population	IT-Eu	75 ± 7	558 (56)	630 (63)	123 (12)	57 (6)	81 (8)
Tadjibaev et al. 2014 [20]	611	General population	RUS-Eu	75 ± 6	441 (72)	567 (93)	87 (14)	91 (15)	484 (79)
Verghese et al. 2012 [21]	567	General population	USA-Am	80 ± 5	345 (61)	355 (63)	93 (16)	56 (10)	69 (12)
Volpato et al. 2011 [22]	87	Hospitalized	IT-Eu	77 ± 6	43 (49)	77 (89)	21 (24)	11 (13)	17 (20)

*SD* standard deviation, *DM* diabetes mellitus, *CVA* cerebrovascular accident, *CVD* cardiovascular disease, *y* years, *Am* America, *Eu* Europe, *As* Asia, *IT* Italy, *BE* Belgium, *FR* France, *RUS* Russia

^a^Injection drug user

### All-cause mortality

**Fig. 2:**
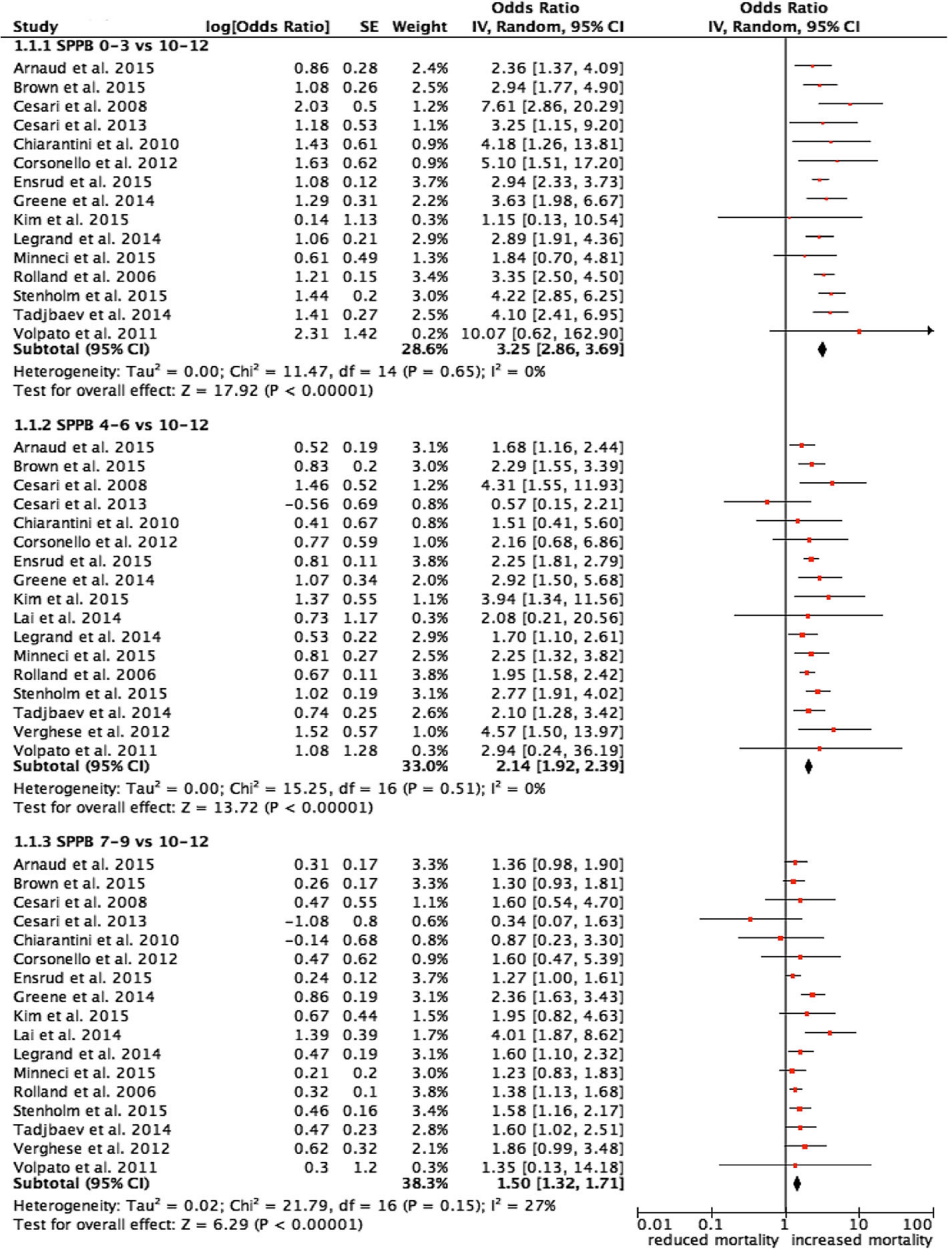
Forest plot of the relation between SPPB and all-cause mortality. Data are displayed for each available study. Error bars represent 95% confidence intervals. *SE* standard error, *CI* confidence interval, *SPPB* Short Physical Performance Battery

Overall, 3237 deaths (19.5%) were recorded. The length of follow-up ranged from a minimum of 1 year to a maximum of 11 years (median 4.5 years) (Additional file 1: eTable 2). As compared to an SPPB score of 10–12, scores of 7–9, 4–6, and 0–3 were associated with greater and progressively increasing risks of all-cause mortality (Fig. 2). After adjustment for age, sex, and body mass index, we found an OR of 1.50 (95%CI 1.32–1.71) for SPPB scores 7–9, OR 2.14 (95%CI 1.92–2.39) for SPPB scores 4–6, and OR 3.25 (95%CI, 2.86–3.79) for SPPB scores 0–3 (Fig. 2). The %AR (95% CI) of death attributed to poor functional performance was 33% (24–42%), 53% (48–58%), and 69% (65–73%), respectively. Heterogeneity, expressed as *I* ^2^%, was insignificant for the comparison between SPPB scores 0–3 and 4–6 versus 10–12, whereas it was low between SPPB scores 7–9 versus 10–12.

### Additional analyses

Subgroup analyses demonstrated that after stratification of the studies for age, type of population, geographic area, and follow-up length, the association between SPPB and all-cause mortality remained highly consistent, with no statistical significance of the interaction terms (Table 2). Random effects meta-regression disclosed no significant association between confounding factors (previous cardiovascular disease, cerebrovascular disease, diabetes, hypertension, age, and sex) for SPPB scores 0–3 or 4–6 versus 10–12 and the risk of all-cause mortality (Additional file 1: eTable 3). In contrast, the OR for all-cause mortality with SPPB scores 7–9 was higher in younger populations, diabetics, and men (Additional file 1: eTable 3, eFigure 2).

**Table 2:** Subgroup analyses after stratification for mean age, population, geographic area, and follow-up length

	*N*	SPPB 0–3 vs. SPPB 10–12			*N*	SPPB 4–6 vs. SPPB 10–12			*N*	SPPB 7–9 vs. SPPB 10–12		
	15	OR (95% CI)	*I* ^2^%	*p*	17	OR (95% CI)	*I* ^2^%	*p*	17	OR (95% CI)	*I* ^2^%	*p*
Mean age				0.37				0.19				0.24
≤75	7	3.54 (2.81–4.46)	0		8	2.40 (1.97–2.93)	0		8	1.65 (1.27–2.16)	59	
>75	8	3.12 (2.67–3.65)	0		9	2.04 (1.79–2.33)	0		9	1.38 (1.22–1.57)	0	
Population				0.36				0.32				0.91
General population	10	3.27 (2.86–3.75)	0		11	2.22 (1.98–2.49)	0		11	1.48 (1.32–1.65)	9	
Outpatients	2	2.53 (1.56–4.12)	0		3	1.45 (0.83–2.52)	14		3	1.48 (0.54–4.08)	80	
Hospitalized	3	4.93(2.18–11.14)	0		3	1.94 (0.85–4.41)	0		3	1.23 (0.53–2.85)	0	
Geographic area				0.40				0.24				0.37
Europe	11	3.44 (2.91–4.07)	0		11	2.02 (1.74–2.33)	3		11	1.42 (1.25–1.60)	0	
Asia	1	1.15(0.13–10.54)	–		1	3.94(1.34–11.56)	–		1	1.95 (0.82–4.63)	–	
North America	3	3.01(2.46–3.69)	0		5	2.34 (1.96–2.80)	0		5	1.79 (1.26–2.55)	73	
Follow-up length				0.49				0.30				0.94
≤1 year	3	4.22 (1.97–9.02)	0		4	1.42 (0.65–3.10)	0		4	1.51 (0.50–4.59)	64	
>1 year	12	3.22 (2.83–3.69)	0		13	2.16 (1.94–2.41)	0		13	1.45 (1.32–1.60)	0	
				0.72				0.77				0.84
≤5 years	10	3.30 (2.82–3.86)	0		11	2.13 (1.86–2.43)	0		11	1.53 (1.23–1.91)	49	
>5 years	5	3.13 (2.48–3.96)	5		6	2.22 (1.73–2.85)	35		6	1.49 (1.27–1.75)	0	

*SPPB* Short Physical Performance Battery, *N* number of studies analyzed for subgroup

### Publication bias

According to graphical evaluation of funnel plots, Begg and Mazumdar rank correlation, and Egger’s regression intercept, there was no evidence of publication bias (Additional file 1: eTable 4 and eFigure 1A-C).

## Discussion

Our meta-analysis suggests that poor performance on the SPPB is associated with an increased risk of all-cause mortality in a dose-response manner. These findings were consistent among community-based subjects and both inpatients and outpatients, and across different geographical areas, age groups, and durations of follow-up.

In the older population, self-reported functional limitation is a well-established independent risk factor for disability, morbidity, hospital admission for any cause, and mortality [3]. Objective measures of physical performance may be more likely to capture the integrated and multisystemic effects of aging, comorbidity, disease severity, malnutrition, motivation, and cognition on the health status of older persons. The SPPB is a simple test developed for assessing lower extremity function. It includes three different assessments (walking speed, chair stand, and balance time) [3, 4]. This test might be considered a non-specific but highly sensitive indicator of global health status and also an indicator of vulnerability [38], reflecting several underlying physiological impairments [39].

To the best of our knowledge, this is the first meta-analysis with an adequate sample size to definitively study the relationship between SPPB score and all-cause mortality. We found an independent association between poor performance on SPPB and all-cause mortality. As expected, the association between SPPB score and all-cause mortality was more pronounced at lowest scores (0–3 and 4–6 versus 10–12). Nevertheless, a 7–9 SPPB score predicted increased all-cause mortality compared to a score of 10–12. It is noteworthy that meta-regression analysis revealed that, in the group of subjects with SPPB scores 7–9, a higher risk of death was seen in males, diabetics, and younger persons.

Previous studies have suggested an association between measures of physical performance and all-cause mortality [40, 41]. In particular, two worthy meta-analyses showed that walking speed, chair stand, and balance time (each tested singularly) were able to discriminate those at heightened risk of mortality in community-dwelling older adults [40, 41]. Our meta-analysis extends these findings into a broader range of ages, clinical settings, and geographical areas. As compared to single tests, SPPB gives a more thorough evaluation of lower limb physical capability, and it could permit a better discrimination of subjects with poor physical function. At the same time, the application of the full SPPB compared to the single part of this test, such as gait speed, is more time-consuming. Future studies are needed to assess if the application in clinical practice of SPPB is superior to the application of gait speed alone in the prediction of mortality, considering also the costs for health care. In effect, one of the limits of the application of SPPB in daily clinical practice is related to the chronic limitation of the resources in the primary care setting. This problem is dual. Firstly, the systematic application of SPPB to elderly patients requires qualified, properly trained personnel. Secondly, the application of self-reported physical function could be a possible alternative, but it is still not known if this assessment could be considered reliable in prediction of mortality.

Our work strongly supports the role of SPPB scores as a marker for risk stratification. This information might eventually support the development of adapted and personalized care offered to older persons. Considering the strong association with all-cause mortality, information on SPPB might suggest the application of different diagnostic and therapeutic strategies tailoring the more aggressive and intensive interventions to elderly patients with low physical performance. Randomized trials are warranted to test whether adoption of SPPB as a prognostic indicator by health systems reduces adverse health-related outcomes or reduces health care costs. For example, use of SPPB may improve choice of post-acute care setting, thereby reducing risk of short-term re-hospitalization and may better identify subsets of older patients unlikely to benefit from invasive surgical procedures. Alternatively, SPPB could be helpful as a surrogate endpoint of all-cause mortality in trials needing to quantify benefit and health improvements of specific treatments or rehabilitation programs. For example, the Lifestyle Interventions and Independence for Elders (LIFE) pilot study demonstrated that a structured physical activity program is able to increase the SPPB score [42]. Subsequently, the investigators showed in a larger randomized trial that a moderate-to-intense program of physical activity reduces disability [3].

### Study limitations

Our results suffer from those limitations that are inherent to all meta-analytic techniques including particularly heterogeneity in populations and variable endpoint definitions across studies. We could analyze data only from authors who replied to our request and, even if statistical analyses do not show the presence of publication bias, this could not be completely excluded. Secondly, we decided to report SPPB score in classes (0–3, 4–6, 7–9, 10–12) and not as a continuous variable. Finally, we only evaluated the association between SPPB and mortality. Additional studies are needed to show that adoption of SPPB into a prediction model improves discrimination of mortality and to evaluate its clinical utility in the practice setting. Nevertheless, this is a meta-analysis on a large sample, including more than 16,000 patients. Our protocol has been prespecified and registered on a public platform (PROSPERO), and the collaboration between authors allowed us to obtain highly standardized data.

## Conclusions

In the present collaborative meta-analysis, a SPPB value less than 10 predicts all-cause mortality. This finding is consistent across different clinical settings, geographical areas, ages, and follow-up lengths.

## Additional file

Additional file 1: Short Physical Performance Battery and all-cause Mortality: Systematic Review and Meta-analysis. **eTable 1**. New-Castle Ottawa Scale for quality assessment. **eTable 2**. Source for follow-up of all the studies included in the meta-analysis. **eTable 3.** Meta-regression analyses considering population characteristics of each study included in the meta-analysis. **eTable 4**. Assessment of publication bias. **eTable 5**. PRISMA checklist. **eFigure 1**. Funnel plot and Trim and Fill analysis. **A**. Relation between SPPB 0-3 vs 10-12 and all-cause mortality. **B**. Relation between SPPB 4-6 vs 10-12 and all-cause mortality. **C**. Relation between SPPB 7-9 vs 10-12 and all-cause mortality. **eFigure 2**. Scatter Plot of meta-regression analysis for female sex, diabetes mellitus and age and relation between SPPB 7-9 vs 10-12 and all-cause death. (DOCX 146 kb)
