# Supplementary material for: Short Physical Performance Battery and all-cause mortality: systematic review and meta-analysis
Source: BMC Med. 2016 Dec 22;14:215. doi: 10.1186/s12916-016-0763-7 (PMC5178082; doi:10.1186/s12916-016-0763-7)
Supplement: Additional file 1: — Short Physical Performance Battery and all-cause Mortality: Systematic Review and Meta-analysis. eTable 1. New-Castle Ottawa Scale for quality assessment. eTable 2. Source for follow-up of all the studies included in the meta-analysis. eTable 3. Meta-regression analyses considering population characteristics of each study included in the meta-analysis. eTable 4. Assessment of publication bias. eTable 5. PRISMA checklist. eFigure 1. Funnel plot and Trim and Fill analysis. A. Relation between SPPB 0-3 vs 10-12 and all-cause mortality. B. Relation between SPPB 4-6 vs 10-12 and all-cause mortality. C. Relation between SPPB 7-9 vs 10-12 and all-cause mortality. eFigure 2. Scatter Plot of meta-regression analysis for female sex, diabetes mellitus and age and relation between SPPB 7-9 vs 10-12 and all-cause death. (DOCX 146 kb) [file 12916_2016_763_MOESM1_ESM.docx]

**Short Physical Performance Battery and all-cause Mortality: Systematic Review and Meta-analysis.**

**Supplemental online material**

**Index**

**Search strategy details consistent with Medline search**

**eTable 1: New-Castle Ottawa Scale for quality assessment**

**eTable 2: Source for follow-up of all the studies included in the meta-analysis**

**eTable 3: Meta-regression analyses considering population characteristics of each study included in the meta-analysis**

**eTable 4: Assessment of publication bias**

**eTable 5: PRISMA checklist**

**eFigure 1: Funnel plot and Trim and Fill analysis.**

A: Relation between SPPB 0-3 vs 10-12 and all-cause mortality.

B: Relation between SPPB 4-6 vs 10-12 and all-cause mortality.

C: Relation between SPPB 7-9 vs 10-12 and all-cause mortality.

**eFigure 2: Scatter Plot of meta-regression analysis for female sex, diabetes mellitus and age and relation between SPPB 7-9 vs 10-12 and all-cause death.**

**Search strategy details consistent with Pubmed**

((short[All Fields] AND ("physical examination"[MeSH Terms] OR ("physical"[All Fields] AND "examination"[All Fields]) OR "physical examination"[All Fields] OR "physical"[All Fields]) AND performance[All Fields] AND battery[All Fields]) OR SPPB[All Fields] OR (("lower extremity"[MeSH Terms] OR ("lower"[All Fields] AND "extremity"[All Fields]) OR "lower extremity"[All Fields] OR ("lower"[All Fields] AND "limb"[All Fields]) OR "lower limb"[All Fields]) AND strength[All Fields]) OR (("posture"[MeSH Terms] OR "posture"[All Fields] OR "standing"[All Fields]) AND ("Balance"[Journal] OR "balance"[All Fields])) OR (("walking"[MeSH Terms] OR "walking"[All Fields]) AND "speed"[All Fields]) OR (chair[All Fields] AND stand[All Fields])) AND (("mortality"[Subheading] OR "mortality"[All Fields] OR "mortality"[MeSH Terms]) OR ("death"[MeSH Terms] OR "death"[All Fields])).

**eTable 1: New-Castle Ottawa Scale (NOS) for quality assessment**

| **References** | **NOS score (maximum 6)** | **Selection 1** | **Selection 3** | **Selection 4** | **Outcome 1** | **Outcome 2** | **Outcome 3** |
| --- | --- | --- | --- | --- | --- | --- | --- |
| ***Arnau et al. 2016*** | 5 | a* | a* | a* | b* | a* | d |
| ***Brown et al. 2015*** | 5 | b* | a* | a* | b* | a* | d |
| ***Cesari et al. 2008*** | 5 | a* | a* | a* | b* | a* | d |
| ***Cesari et al. 2013*** | 5 | c | a* | a* | b* | a* | a* |
| ***Chiarantini et al. 2010*** | 5 | b* | a* | a* | b* | a* | d |
| ***Corsonello et al. 2012*** | 6 | a* | a* | a* | b* | a* | a* |
| ***Ensrud et al. 2015*** | 5 | a* | a* | a* | b* | a* | d |
| ***Greene et al. 2014*** | 5 | b* | a* | a* | b* | a* | d |
| ***Kim et al. 2015*** | 6 | a* | a* | a* | b* | a* | b* |
| ***Lai et al. 2014*** | 5 | b* | a* | a* | b* | a* | d |
| ***Legrand et al. 2014*** | 6 | a* | a* | a* | b* | a* | a* |
| ***Minneci et al. 2015*** | 5 | a* | a* | a* | b* | a* | d |
| ***Rolland et al. 2006*** | 6 | a* | a* | a* | b* | a* | a* |
| ***Tadjibaev et al. 2014*** | 5 | a* | a* | a* | d | a* | a* |
| ***Stenholm et al. 2016*** | 5 | a* | a* | a* | b* | a* | d |
| ***Verghese et al. 2012*** | 6 | a* | a* | a* | b* | a* | b* |
| ***Volpato et al. 2011*** | 6 | b* | a* | a* | b* | a* | b* |

*: value corresponding to 1 point for score

Because of the design of the studies, we decided to use the NOS scale for cohort studies, but not considering the section for “Comparability” and question 2 in the section “Selection” (“selection of the non exposed cohort”). Letters indicate the answer to questions as indicated in the original form of the NOS scale for cohort studies. For the full version of the NOS scale : <http://www.ohri.ca/programs/clinical_epidemiology/oxford.asp>.

**eTable 2: Source for follow-up of all the studies included in the meta-analysis**

| **References** | **Number of deaths** | **Follow-up length**  **(years)** | **Source for mortality data** |
| --- | --- | --- | --- |
| ***Arnau et al. 2016*** | 209 | 10 | Family, nursing home, attending physician, Mortality Register |
| ***Brown et al. 2015*** | 315 | 11 | National death index database |
| ***Cesari et al. 2008*** | 23 | 1.8 | Medical reports |
| ***Cesari et al. 2013*** | 71 | 1 | National death registry, general practitioner |
| ***Chiarantini et al. 2010*** | 61 | 1.2 | Administrative database and phone interview |
| ***Corsonello et al. 2012*** | 67 | 1 | Patients, relatives, caregiver via phone call |
| ***Ensrud et al. 2015*** | 749 | 4.9 | Death certificate |
| ***Greene et al. 2014*** | 165 | 5 | Death certificates, National Death Index |
| ***Kim et al. 2015*** | 59 | 6 | Phone interview |
| ***Lai et al. 2014*** | 36 | 1 | Patient’s electronics health record |
| ***Legrand et al. 2014*** | 129 | 5.2 | Questionnaire filled out by General Pratictioner |
| ***Minneci et al. 2015*** | 141 | 4 | City registry office |
| ***Rolland et al. 2006*** | 754 | 3.8 | Phone call to proxies and primary care |
| ***Stenholm et al. 2016*** | 363 | 6 | Mortality general registry by Tuscany Region and death certificate |
| ***Tadjibaev et al. 2014*** | 35 | 2.5 | NS |
| ***Verghese et al. 2012*** | 49 | 6.8 | Visits and phone interview, social security death index |
| ***Volpato et al. 2011*** | 11 | 1 | Phone interview |

NS= not specified

**eTable 3: Meta-regression analyses considering population characteristics of each study included in the meta-analysis**

|  | **N°** | **Beta** | **p** |
| --- | --- | --- | --- |
| ***SPPB 0-3 vs SPPB 10-12*** |  |  |  |
| *Age* | 15 | -0.01 | 0.38 |
| *Hypertension* | 12 | 0 | 0.52 |
| *Diabetes* | 13 | 0.00 | 0.81 |
| *Female sex* | 15 | -0.00 | 0.45 |
| *CVA* | 11 | -0.10 | 0.45 |
| *CVD* | 13 | 0.00 | 0.73 |
| ***SPPB 4-6 vs SPPB 10-12*** |  |  |  |
| *Age* | 17 | -0.01 | 0.34 |
| *Hypertension* | 14 | 0.00 | 0.86 |
| *Diabetes* | 15 | 0 | 0.72 |
| *Female sex* | 17 | -0.00 | 0.32 |
| *CVA* | 13 | 0.01 | 0.38 |
| *CVD* | 15 | -0.00 | 0.37 |
| ***SPPB 7-9 vs SPPB 10-12*** |  |  |  |
| *Age* | 17 | **-0.02** | **0.008** |
| *Hypertension* | 14 | -0.00 | 0.97 |
| *Diabetes* | **15** | **0.02** | **0.03** |
| *Female sex* | **17** | **-0.01** | **0.02** |
| *CVA* | 13 | -0.00 | 0.79 |
| *CVD* | 15 | 0.00 | 0.50 |

CVA: cerebral-vascular accident; CVD: cardiovascular disease; SPPB: short physical performance battery; N°: number of study analyzed. In **BOLD** statistical significant analyses.

**eTable 4: Assessment of publication bias**

|  | **SPPB 0-3 vs 10-12** | | **SPPB 4-6 vs 10-12** | | **SPPB 7-9 vs 10-12** | |
| --- | --- | --- | --- | --- | --- | --- |
| **Eggers linear regression test** |  | **p** |  | **p** |  | **p** |
| *Intercept* | 0.32 | 0.494 | 0.28 | 0.519 | 0.34 | 0.546 |
| *T* | 0.70 |  | 0.66 |  | 0.62 |  |
| **Begg and Mazumdar’s rank correlation test** |  |  |  |  |  |  |
| *Z value for Kendall’s tau* | 0.84 | 0.40 | 0.00 | 1.00 | -0.25 | 0.805 |
| **Trim and fill test** |  |  |  |  |  |  |
| *OR estimated* | 3.25 | <0.0001 | 2.09 | <0.0001 | 1.50 | <0.0001 |
| *N° trimmed studies* | 1 |  | 3 |  | 0 |  |

SPPB: Short Physical Performance Battery

**eTable 5: PRISMA Checklist**

| **Section/topic** | **#** | **Checklist item** | **Reported on page #** |
| --- | --- | --- | --- |
| **TITLE** | | |  |
| Title | 1 | Identify the report as a systematic review, meta-analysis, or both. | 1 |
| **ABSTRACT** | | |  |
| Structured summary | 2 | Provide a structured summary including, as applicable: background; objectives; data sources; study eligibility criteria, participants, and interventions; study appraisal and synthesis methods; results; limitations; conclusions and implications of key findings; systematic review registration number. | 6 |
| **INTRODUCTION** | | |  |
| Rationale | 3 | Describe the rationale for the review in the context of what is already known. | 8 |
| Objectives | 4 | Provide an explicit statement of questions being addressed with reference to participants, interventions, comparisons, outcomes, and study design (PICOS). | 8 |
| **METHODS** | | |  |
| Protocol and registration | 5 | Indicate if a review protocol exists, if and where it can be accessed (e.g., Web address), and, if available, provide registration information including registration number. | 9 |
| Eligibility criteria | 6 | Specify study characteristics (e.g., PICOS, length of follow-up) and report characteristics (e.g., years considered, language, publication status) used as criteria for eligibility, giving rationale. | 10 |
| Information sources | 7 | Describe all information sources (e.g., databases with dates of coverage, contact with study authors to identify additional studies) in the search and date last searched. | 9-11 |
| Search | 8 | Present full electronic search strategy for at least one database, including any limits used, such that it could be repeated. | 9, e-page 3 |
| Study selection | 9 | State the process for selecting studies (i.e., screening, eligibility, included in systematic review, and, if applicable, included in the meta-analysis). | 10 |
| Data collection process | 10 | Describe method of data extraction from reports (e.g., piloted forms, independently, in duplicate) and any processes for obtaining and confirming data from investigators. | 10 |
| Data items | 11 | List and define all variables for which data were sought (e.g., PICOS, funding sources) and any assumptions and simplifications made. | 10 |
| Risk of bias in individual studies | 12 | Describe methods used for assessing risk of bias of individual studies (including specification of whether this was done at the study or outcome level), and how this information is to be used in any data synthesis. | 11 |
| Summary measures | 13 | State the principal summary measures (e.g., risk ratio, difference in means). | 11 |
| Synthesis of results | 14 | Describe the methods of handling data and combining results of studies, if done, including measures of consistency (e.g., I^2^) for each meta-analysis. | 11 |

Page 1 of 2

| **Section/topic** | **#** | **Checklist item** | **Reported on page #** |
| --- | --- | --- | --- |
| Risk of bias across studies | 15 | Specify any assessment of risk of bias that may affect the cumulative evidence (e.g., publication bias, selective reporting within studies). | 11-12 |
| Additional analyses | 16 | Describe methods of additional analyses (e.g., sensitivity or subgroup analyses, meta-regression), if done, indicating which were pre-specified. | 11-12 |
| **RESULTS** | | |  |
| Study selection | 17 | Give numbers of studies screened, assessed for eligibility, and included in the review, with reasons for exclusions at each stage, ideally with a flow diagram. | 13 |
| Study characteristics | 18 | For each study, present characteristics for which data were extracted (e.g., study size, PICOS, follow-up period) and provide the citations. | 13-14  Table 1 |
| Risk of bias within studies | 19 | Present data on risk of bias of each study and, if available, any outcome level assessment (see item 12). | eTable1 |
| Results of individual studies | 20 | For all outcomes considered (benefits or harms), present, for each study: (a) simple summary data for each intervention group (b) effect estimates and confidence intervals, ideally with a forest plot. | Figure 2 |
| Synthesis of results | 21 | Present results of each meta-analysis done, including confidence intervals and measures of consistency. | Figure 2, Table 2 |
| Risk of bias across studies | 22 | Present results of any assessment of risk of bias across studies (see Item 15). | 15, eTable 4, eFigure 2 |
| Additional analysis | 23 | Give results of additional analyses, if done (e.g., sensitivity or subgroup analyses, meta-regression [see Item 16]). | 14-15,  Table2, eTable3, eFigure1 |
| **DISCUSSION** | | |  |
| Summary of evidence | 24 | Summarize the main findings including the strength of evidence for each main outcome; consider their relevance to key groups (e.g., healthcare providers, users, and policy makers). | 16-18 |
| Limitations | 25 | Discuss limitations at study and outcome level (e.g., risk of bias), and at review-level (e.g., incomplete retrieval of identified research, reporting bias). | 18 |
| Conclusions | 26 | Provide a general interpretation of the results in the context of other evidence, and implications for future research. | 18 |
| **FUNDING** | | |  |
| Funding | 27 | Describe sources of funding for the systematic review and other support (e.g., supply of data); role of funders for the systematic review. | 20 |

*From:*  Moher D, Liberati A, Tetzlaff J, Altman DG, The PRISMA Group (2009). Preferred Reporting Items for Systematic Reviews and Meta-Analyses: The PRISMA Statement. PLoS Med 6(6): e1000097. doi:10.1371/journal.pmed1000097

For more information, visit: **www.prisma-statement.org**.

Page 2 of 2

**Figure 1A: Funnel plot and Trim and Fill analysis of the relation between SPPB 0-3 vs 10-12 and all-cause mortality.**

White dots: observed values; black dots: estimated values. SPPB: short physical performance battery


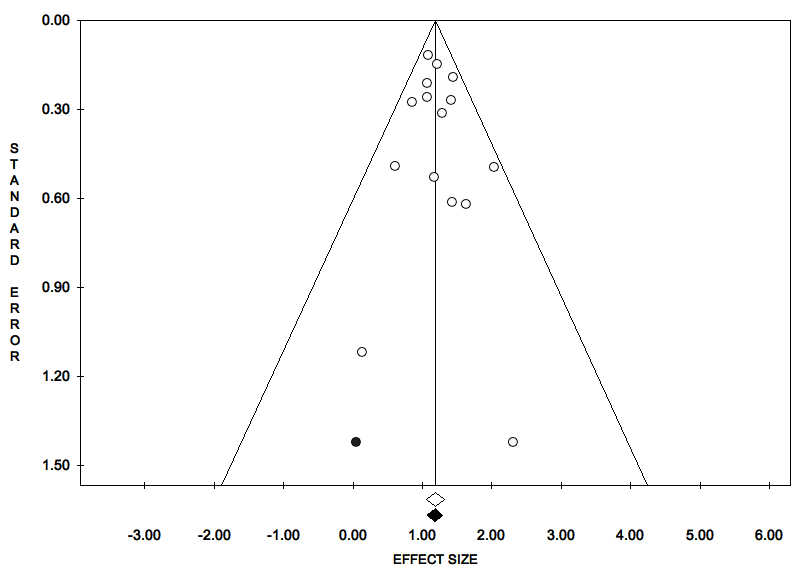


**Figure 1B: Funnel plot and Trim and Fill analysis of the relation between SPPB 4-6 vs 10-12 and all-cause mortality**

White dots: observed values; black dots: estimated values. SPPB: short physical performance battery


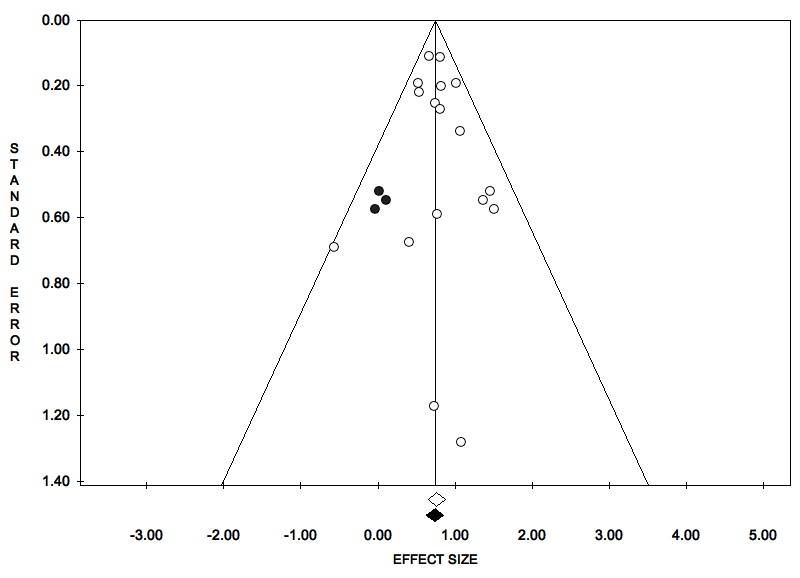


**Figure 1C: Funnel plot and Trim and Fill analysis of the relation between SPPB 4-6 vs 10-12 and all-cause mortality**

White dots: observed values; black dots: estimated values. SPPB: short physical performance battery


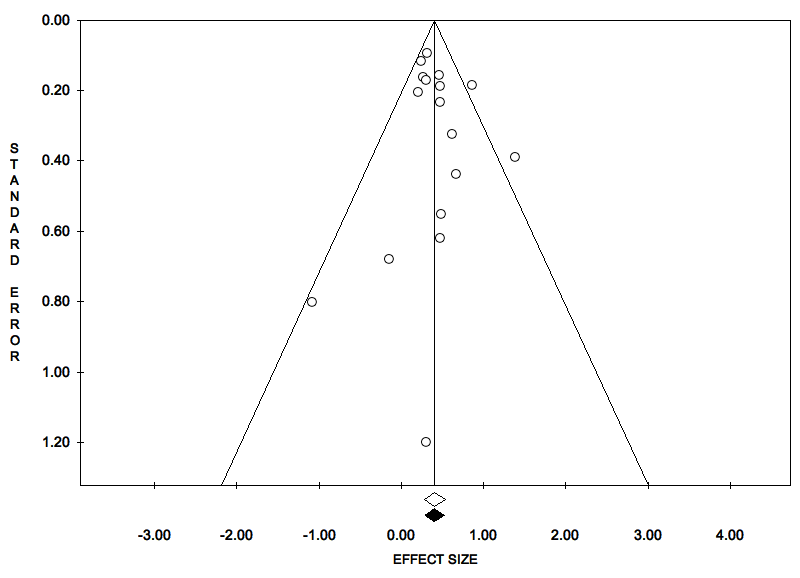


**Figure 2: scatter Plot of meta-regression analysis for female sex, diabetes mellitus and age and relation between SPPB 7-9 vs 10-12 and all-cause death.**

Effect size is referred to Ln[Odds Ratio]. Data on “x axis” are expressed as percentage for female sex and diabetes mellitus, as continuous variable for age. DM: diabetes mellitus.

**
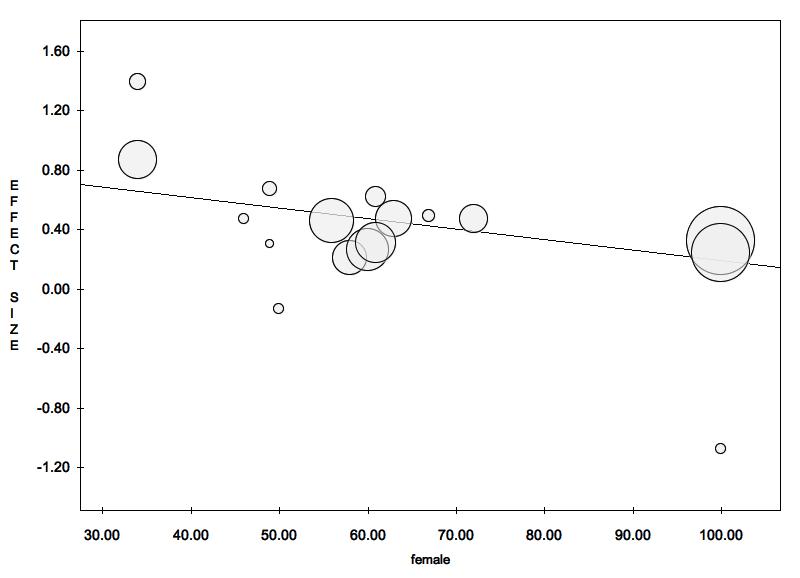
**

**
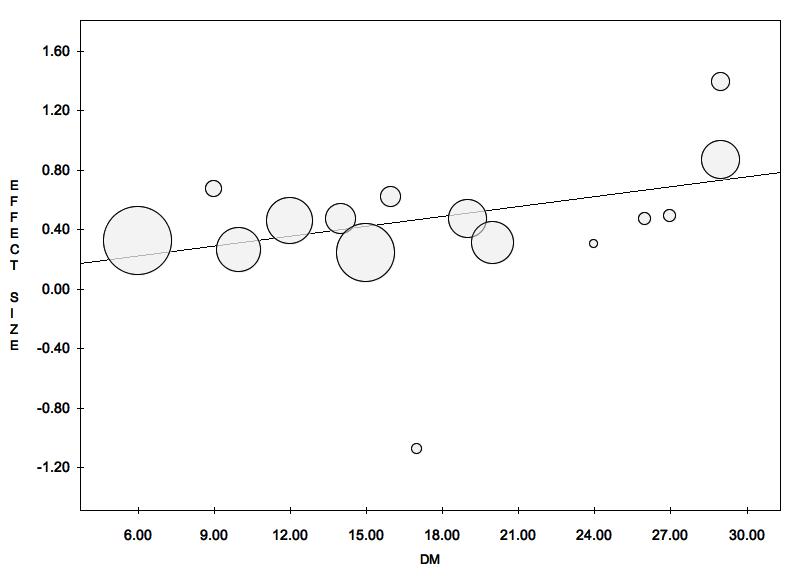
**

**
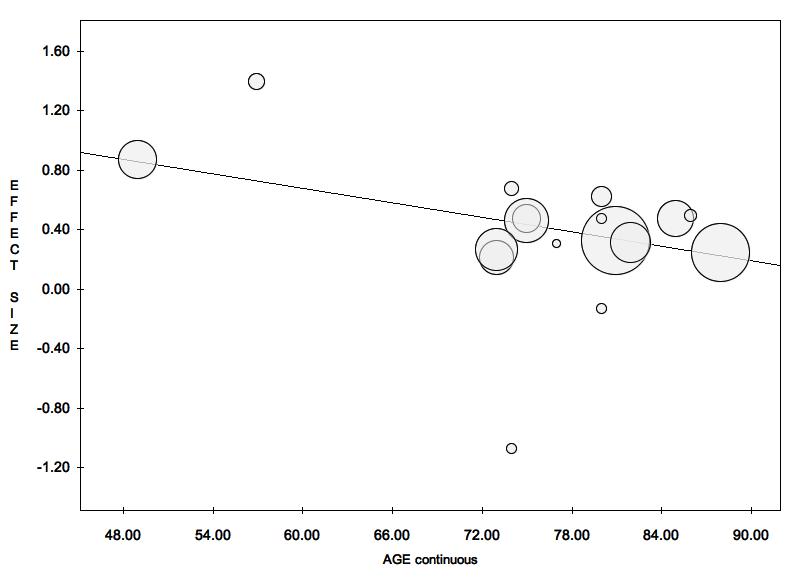
**
